# Is the network of heterosexual contact in Japan scale free?

**DOI:** 10.1371/journal.pone.0221520

**Published:** 2019-08-27

**Authors:** Hiromu Ito, Kazuhiro Tamura, Takayuki Wada, Taro Yamamoto, Satoru Morita

**Affiliations:** 1 Department of International Health, Institute of Tropical Medicine, Nagasaki University, Nagasaki, Japan; 2 Department of Environmental Sciences, Zoology, University of Basel, Basel, Switzerland; 3 Department of Environment and Energy Systems, Graduate School of Science and Technology, Shizuoka University, Hamamatsu, Shizuoka, Japan; 4 School of Tropical Medicine and Global Health, Nagasaki University, Nagasaki, Japan; 5 Department of Mathematical and Systems Engineering, Graduate School of Integrated Sciences and Technology, Shizuoka University, Hamamatsu, Shizuoka, Japan; Weill Cornell Medical College - Qatar, QATAR

## Abstract

Elucidation of the structure of human sexual networks is not only an interesting topic in the area of social networks but also an important clue for understanding the spreading risk of sexually transmitted infections (STIs). Some previous studies have indicated that sexual networks are scale free, while others have suggested that they are not. We conducted a Web-based survey on sexual contact in Japan to collect data on cumulative (total) heterosexual partners and the number of recent (in the last three or previous three months) heterosexual partners. To determine whether the number of heterosexual contacts in Japan has a power-law tail, we used maximum likelihood fitting methods and Kolmogorov-Smirnov tests. For confirmation, we also used the Akaike information criterion (AIC) and the Bayesian information criterion (BIC). Our results indicate that the distributions of the number of sexual partners in Japan have power-law tails.

## Introduction

Understanding the structure of human sexual contact is important for elucidating the risk factors for sexually transmitted infections (STIs) and for understanding the relationship between the social structure and infection dynamics of STIs [1]. Some previous studies have indicated that networks of heterosexual contact have scale-free properties [2–4], while others have suggested that they do not [5,6]. Here, a scale-free network is defined as a network in which the number of sexual partners of a given individual has a power-law tail, which was calculated as follows:

$$P(k)\propto k^{-\alpha },$$ (1)

where *k* and *α* represent the number of sexual partners and the power-law exponent, respectively. The heterogeneity of human sexual contacts is increased when the power-law exponent, *α*, is decreased. It is well known that if the sexual contact network is a scale-free network with *α*≤3, the spreading threshold of STIs in the network approaches 0 in the limit of a large population size [7–9]. In this case, STIs can survive even if the sexual transmission rate is extremely low. Thus, examining the scale-free properties of sexual networks is an important issue in public health.

Handcock and Jones [5] assessed the model fit with the Akaike information criterion (AIC) and Bayesian information criterion (BIC) and showed that for sexual contacts in the USA, Sweden and Uganda, there were cases in which a negative binominal distribution fit the data better than a power-law distribution. In addition, Hamilton et al. [6] reevaluated various sexual network data, including data from England and Zimbabwe, and suggested that power-law models do not always fit the data best. In these previous works, interview surveys were conducted at the regional/local level because large-scale interview surveys could not be easily conducted due to time limitations, financial costs and privacy concerns [10,11]. Thus, the structural characteristics of sexual networks remain a mystery in the general setting.

Currently, Web-based surveys (Internet surveys) have a great influence on survey research [12,13]. Web-based surveys do not have a long history, but they have drawn attention in various research fields because the cost of Web surveys is considerably less than the cost of data collection via phone or face-to-face interviews [14–17]. In addition, Web-based surveys are easy to prepare and execute, allowing more data to be collected in a short period of time. Therefore, Web-based surveys are increasingly popular in health survey research [11,12]. In the past, it was thought that Web-based surveys had a strong sampling bias (especially with regard to age and sex) because there were some people who did not use Internet [11]. As of 2016, however, most (83.5%) of the people in Japan regularly use the Internet [18]. Thus, we can expect that the sampling bias of Web surveys will decrease as Internet usage increases. Furthermore, since participants can answer a questionnaire in a Web-based survey without having direct contact with researchers, it is likely they will be more willing to share their personal sexual information than those that complete face-to-face interviews. Notably, conventional face-to-face interview surveys are impractical in Japan. The social advancement of women has resulted in empty households when visiting to administer surveys. In addition, due to the revision of the Basic Resident Register Act in 2006, random sampling from the Resident Registration System of Japan has become illegal, except in the case of governmental studies. For these reasons, Web surveys are the only practical survey methods that can reach a large number of people in Japan, even though some sampling bias may be present.

In this study, we conducted a Web-based survey on sexual contact in Japan. A total of 5,000 individuals (2,500 males and 2,500 females) who were between the ages of 18 and 88 years participated. We asked about the participants’ cumulative (total) number of heterosexual partners and the number of recent (last three months) heterosexual partners. We fit a power-law distribution to the data and compared it to the fit of geometrical and truncated power-law distributions through goodness-of-fit tests based on the Kolmogorov-Smirnov statistic and likelihood ratios [19–21]. In addition, we used the AIC and BIC approaches to estimate whether a negative binomial distribution or a power-law distribution fit the data better [5]. Concerning homosexual contacts, we asked only about the occurrence of a same-sex sexual experience rather than the frequency. It is thought that only a small percentage of people in Japan have had homosexual contact [22].

## Materials and methods

### Recruitment

An Internet survey entitled “Survey on the Japanese sexual network” was administered to a Japanese population in May 2018. The investigation period was seven days, from May 15 to 21, 2018. For this survey, an Internet survey company (Cross Marketing Inc.) created the questionnaire webpages based on our study design, which is described later. The company also conducted the data collection. The Internet survey panel included 1.74 million people, which corresponded to approximately 1% of the total population of Japan (126.49 million as of 1 May 2018), who were already registered with the company [23]. For survey participants who registered in advance, the questionnaire and response column were displayed on the website through which the respondents could complete and submit their responses. We extracted 2,500 submissions for each sex by random sampling from all of the samples collected between May 15 and 21, 2018. Finally, we obtained 5,000 responses (2,500 from males and 2,500 from females) from individuals between 18 and 88 years of age. All respondents provided informed consent before completing the questionnaire. The respondents were rewarded with electronic points that can be exchanged for cash, gift certificates, frequent flyer miles and electronic money (e-money) for various services. The exact amount of the electronic point reward is unknown due to the company’s privacy policy. This study was approved by the Ethical Committee of the Institute of Tropical Medicine, Nagasaki University (Approval No. 171207183).

The respondents provided their anonymous personal information (age, sex, etc.) and answered the following three questions:

- How many partners have you had heterosexual contact with in your entire life? (0–999);
- How many partners have you had heterosexual contact with in the last three months? (0–999); and
- Have you experienced sexual contact with a person of the same sex? (YES or NO).

Prior to answering the questions, we clarified that “sexual contact” referred to three types of sexual contact: sexual intercourse, anal sex and oral sex. We asked the respondents to enter a numeric value from 0 to 999 for questions (I) and (II).

### Statistical analysis

We fit the power-law distribution to the data in the region *k*≥*k*_min_ because small values in the data do not follow the power-law distribution. Here, the optimal value of *k*_min_ was selected, which was the value that resulted in the minimal Kolmogorov-Smirnov distance between the data and the fit^21^. We adopted this method to determine the optimal *k*_min_ because the standard AIC and BIC overestimated the value of *k*_min_ due to overfitting in the region *k*<*k*_min_, as shown later (see Clauset et al. [20]). Then, we obtained the estimated value of the power exponent *α* at the calculated optimal value of *k*_min_. To determine whether the power-law distribution provided a good description of the data, we compared it to the fit of other distributions (geometrical and truncated power-law distributions) using goodness-of-fit tests based on the Kolmogorov-Smirnov statistic and calculated log-likelihood ratios between the two candidate distributions and p-values to determine the significance of the results [19–21].

Moreover, we compared the fittings of the power-law distribution and the negative binomial distribution with the AIC and BIC because some previous studies have suggested that a negative binomial distribution gives the best fit for sexual networks across several countries or regions [5,6]. For a sample of *n* people with data *k*_1_,*k*_2_,…,*k*_*n*_, the log-likelihood function L is represented as follows:

$$L(\pi_{0},\pi_{1},\ldots ,\pi_{k_{min}-1},\theta |K_{1}=k_{1},K_{2}=k_{2},\ldots ,K_{n}=k_{n})=\sum_{k=0}^{k_{min}-1}n_{k}\;\log\left(\pi_{k}\right)+(n-\sum_{k=0}^{k_{min}-1}n_{k})\log\left(1-\sum_{k=0}^{k_{min}-1}\pi_{k}\right)+\sum_{k=k_{min}}^{\infty }n_{k}\;\log\left[P(K=k|K\geq k_{min})\right],$$ (2)

where *n*_*k*_ is the observed frequency of network degree *k*, and *n* is the total observations ($n=\sum_{k=0}^{\infty }n_{k}$) [5]. We assumed a model in which *P*(*K* = *k*) = *π*_*k*_ for *k*<*k*_min_ and that follows a power-law distribution or negative binomial distribution for *k*≥*k*_min_. The first, second and third terms of the right-hand side of Eq (2) correspond to *P*(*K* = *k*), *P*(*K*≥*k*_min_), and *P*(*K* = *k*|*K*≥*k*_min_), respectively. The power-law distribution is as follows:

$$P(K=k|K\geq k_{min})=\frac{k^{-\alpha }}{\zeta (\alpha ,k_{min})}$$ (3)

where $\zeta (\alpha ,k_{min})=\sum_{k=0}^{\infty }{\left(k+k_{min}\right)}^{-\alpha }$ is the generalized zeta function. Specifically, we used the shifted negative binomial distribution,

$$P(K=k|K\geq k_{min})\propto p^{k-1}{\left(1-p\right)}^{r}\left(\begin{array}{c} k+r-2\\ k-1 \end{array}\right),$$ (4)

rather than a negative binomial distribution because it was regarded as a better model in a prior study [5,6]. The results in the next section do not change qualitatively if the latter is adopted.

Thus, AIC and BIC were defined as follows:

$$AIC=-2L(\pi_{0},\pi_{1},\ldots ,\pi_{k_{min}-1},\theta |K_{1}=k_{1},K_{2}=k_{2},\ldots ,K_{n}=k_{n})+2d,$$ (5)

$$BIC=-2L(\pi_{0},\pi_{1},\ldots ,\pi_{k_{min}-1},\theta |K_{1}=k_{1},K_{2}=k_{2},\ldots ,K_{n}=k_{n})+\log\left(n\right)\;d,$$ (6)

where *π*_*k*_ = *P*(*K* = *k*) for *k*<*k*_min_, *θ* represents the parameters of the distribution for *K*≥*k*_min_, and *d* is the number of parameters. We set *d* = *k*_min_+1 for the power-law distribution, *d* = *k*_min_+2 for the negative binomial distribution, and *d* = *k*_min_+3 for the truncated power-law distribution. If a model had a relatively smaller AIC or BIC value, it was judged to be good. Using the maximum likelihood method, we obtained another estimated value of the power-law exponent *α*, and we evaluated the confidence interval (CI) by the nonparametric (percentile) bootstrap method [24].

## Results

**Fig 1:**
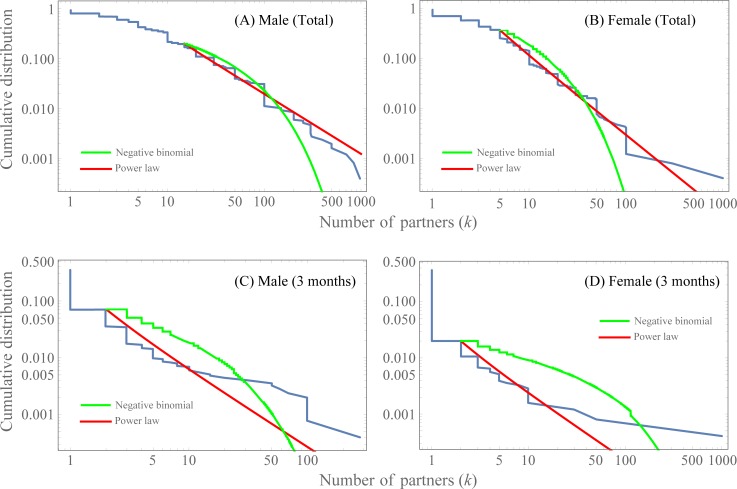
The cumulative distributions of the number of sexual partners. The cumulative distributions for the number of cumulative (total) sexual partners for (**A**) males and (**B**) females, respectively. (**C**) and (**D**) show the cumulative distributions of the number of sexual partners for males and females in the previous three months, respectively. The red and green curves represent the maximum likelihood fitting of the power-law distribution and the negative binomial distribution, respectively. Here, we use *k*_min_ as shown in Table 1. The estimated value and confidence interval of the power-law exponent are given in Table 1.

The data obtained by our Web survey are presented in an MS excel spreadsheet in Supplementary Information (S1 Data and S2 Data), and the age composition is shown in S1 Fig The average ages of the survey participants were 53.8 and 44.9 for males and females, respectively. These values were high for males and low for females compared to the data (44.8 and 48.0 for males and females, respectively) from The Statistics Bureau of Japan. S1 Fig shows that elderly people and young people tended not to participate in the survey. The percentage of males and females who had never had sexual contact (*k* = 0) was 6.6% and 8.2%, respectively, and males and females reported having an average of 13.8 and 5.75 cumulative (total) sexual partners, respectively. Although this type of gap was reported in a previous study [25,26], the current gap may be partially because the male subjects in this survey were relatively older and the female subjects were relatively younger. On the other hand, 64.4% of males and 64.1% of females had experienced no sexual contact in the previous three months. The average number of sexual partners in the previous three months was 0.88 for males and 0.84 for females. Fig 1 shows the cumulative distributions of the number of heterosexual partners for *k*≥1. We show the result of fitting the power-law distribution to the data in Table 1. For the cumulative number of sexual partners, the optimal values of *k*_min_ were provided by 15 and 5 for males and females, respectively, whereas for the number of sexual partners in the previous three months, *k*_min_ = 2 regardless of sex. As shown in Table 1, for all four cases, the estimated values of the power exponent *α* were within the interval between 2 and 3, and the power-law distribution was better than the geometrical distribution. On the other hand, regarding the number of cumulative sexual partners for males, the truncated power-law distribution was fitted significantly better than the power-law distribution. In this case, the truncated power-law distribution is as follows:

**Table 1:** Estimate of the parameters for power-law fitting. The optimal value of k_min_ and the estimated value (1) of the power-law exponent α are derived by minimizing the Kolmogorov-Smirnov distance between the data and the fit.

	*k*_min_	*α* ^(1)^	geo	tpl	*α* ^(2)^
Male (lifetime)	15	2.20	142** (p = 0.000)	−2.49* (p = 0.03)	2.20 (CI: 2.10–2.28)
Female (lifetime)	5	2.54	351** (p = 0.002)	−0.073 (p = 0.70)	2.56 (CI: 2.45–2.65)
Male (3 months)	2	2.24	173** (p = 0.000)	−0.058 (p = 0.73)	2.34 (CI: 2.06–2.56)
Female (3 months)	2	2.11	98.9** (p = 0.002)	−0.087 (p = 0.68)	2.17 (CI: 1.65–2.53)

The columns “geo” and “tpl” are the log-likelihood ratio, comparing the fit of the power-law distribution with that of the geometrical and truncated power-law distributions, respectively. This number is positive if the power-law distribution is favored over the alternative.

The asterisks indicate statistical significance (** p<0.01, * p<0.05). In the last column, the other estimated value (2) of *α* is calculated by the maximum likelihood method, and the CI is evaluated by the nonparametric (percentile) bootstrap method.

$$P(k)\propto k^{-\alpha }e^{-k/k_{c}}$$ (7)

for *k*≥*k*_min_ = 15, where the estimated values of the parameters were *α* = 2.07, and *k*_*c*_ = 906. When we calculated the AIC and BIC, the truncated power-law distribution had an AIC that was 3.14 lower and a BIC that was 2.69 higher than those of the power-law distribution. Thus, we cannot confirm or deny that there was an exponential cutoff value. For the other three cases, there were no significant differences between the truncated power-law distribution and the power-law distribution.

**Fig 2:**
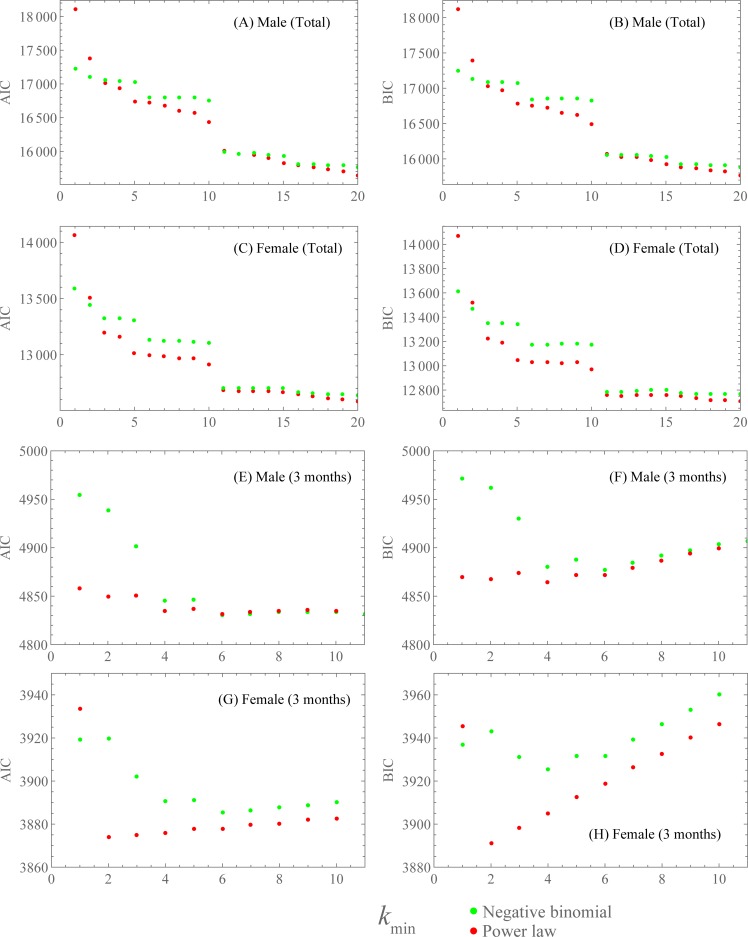
Model selection using the AIC and BIC to compare the fitting of the power-law distribution (red dots) and the shifted negative binomial distribution (green dots) for the number of sexual partners. (**A, C, E, G**) show the AIC, and (**B, D, F, H**) show the BIC of the fitting in a function of *k*_min_. (**A, B**) and (**C, D**) show the fitting for the number of cumulative (total) sexual partners among males and females, respectively. (**E, F**) and (**G, H**) show the fitting for the number of sexual partners among males and females in the previous three months, respectively.

Fig 2A and 2C show the AIC of the fitting for the cumulative number of sexual partners for 1≤*k*_min_≤20. Since the AIC decreased when *k*_min_ increased, we determined the optimal value of *k*_min_ by the AIC. For most values of *k*_min_ (especially *k*_min_ = 15 for males and *k*_min_ = 5 for females), the AIC was less for the power-law distribution than for the negative binominal distribution. The BIC exhibited nearly the same behavior as the AIC, as shown in Fig 2B–2D. These results suggest that the power-law distribution was more suitable than the negative binominal distribution.

**Fig 3:**
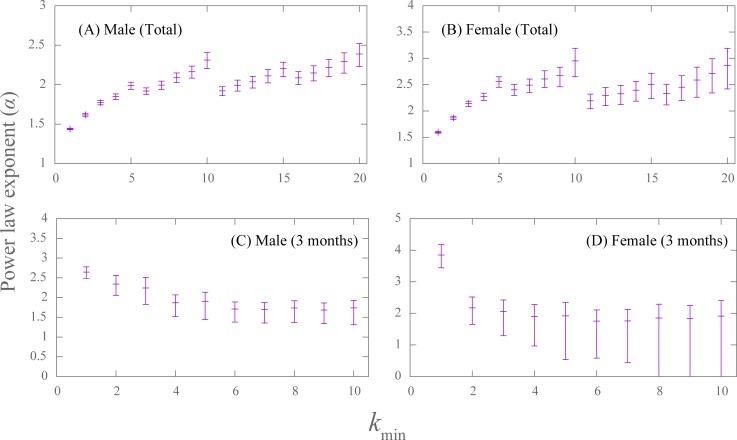
The estimated values of the power-law exponents (*α*) in a function of *k*_min_. (**A**) and (**B**) are cumulative (total) sexual partners for males and females, respectively. (**C**) and (**D**) represent males and females in the previous three months, respectively. The error bars represent the 95% confidence intervals valuated by the nonparametric (percentile) bootstrap method.

Fig 2E–2H show the AIC and BIC of the fitting for the number of sexual partners in the previous three months for 1≤*k*_min_≤10. For males, the AIC and BIC were minimized at *k*_min_ = 6 and *k*_min_ = 4, respectively. These values were overestimated and were higher than the estimated value (*k*_min_ = 2) obtained by using the minimal Kolmogorov-Smirnov distance. For *k*_min_ = 2, the AIC and BIC had lower values for the power-law distribution than for the negative binomial distribution. For females, the AIC and BIC were minimized at *k*_min_ = 2, which coincides with the estimation obtained by using the minimal Kolmogorov-Smirnov distance. For *k*_min_>1, both the AIC and BIC were lower for the power-law distribution than for the negative binominal distribution. This result supports the power-law distribution. In Fig 1, we show the fitting curves for *k*_min_ given in Table 1. In Fig 3, the estimated values of the power-law exponent *α* are plotted as a function of *k*_min_.

The percentages of males and females who had a same-sex sexual experience were 3.6% and 3.0%, respectively; there was no significant difference between the sexes. These numbers were within the range we expected based on previous studies; for example, a Web survey in 2009 estimated that 2.9% of males had same-sex sexual experiences [27], and another Web survey in 2013 reported that 4.1% and 5.1% of males and females, respectively, had same-sex sexual experiences [28].

## Discussion

We studied the sexual contact network in Japan via an Internet survey. To the best of our knowledge, this survey represents the first in Asia on this topic. Our results suggest that the heterosexual network in Japan has a power-law property. However, there is a possibility that a cutoff threshold exists, especially for the number of cumulative sexual partners for males. Additionally, the power-law distribution cannot be entirely accurate due to physiological limitations. The estimated values of the power-law exponents in the Japanese sexual network were less than three and, moreover, less than those found in other countries in previous studies. For example, *α* = 2.48 for males and *α* = 3.1 for females in England, *α* = 3.25 for males and *α* = 4.23 for females in Sweden, *α* = 3.03 for males and *α* = 3.84 for females in the USA, and *α* = 3.07 for males and *α* = 2.51 for females in Zimbabwe [5,6]. This finding suggests the possibility that the spreading risk of STIs in the Japanese population may be higher than previously thought. However, we cannot deny the effect of the survey method on the power-law exponent, as previous studies utilized personal interviews rather than Web-based surveys. Since the current survey is not a face-to-face interview, the respondent is responsible for reporting personal sexual information. Consequently, there is a possibility that some respondents may have entered exaggerated values facetiously or negligent erroneous inputs. In fact, there was a woman who entered the maximum value (i.e., 999) in the text boxes (input boxes) for the number of lifetime cumulative partners and the number of partners in the last 3 months. Therefore, we regarded this respondent as an outlier (S2 Fig and S3 Fig). There is no qualitative difference, and thus, the power-law distribution gives a better fitting. It is difficult to specify criteria setting outliers, because is so the frequency of sexual contact diverse that it is impossible to judge outliers by our common sense. Elimination of a small number of subjects as outliers does not change the qualitative conclusion that the power-law distribution is favorable, even if changes the estimated value of the power-law exponent.

We should note that in some studies, partner numbers were binned into categories in the questionnaire, for example 0, 1, 2–5, 5–10, 10–20, 20–100 partners. In the current study, however, the respondents had to input a numerical value directly. This requirement caused considerable discrepancy between the empirical data and the model because many respondents tended to answer in “round” numbers, such as 5, 10, 20, 50, 100. This discrepancy caused overfitting in the region *k*<*k*_min_;; thus, the AIC and BIC overestimated *k*_min_. To study the effect of rounded numbers, we conducted an additional Web survey in which the subjects did not enter the number of sexual partners directly but selected from the following categories: 1, 2, 3, 4, 5–10, 11–20, 21–50, 51–100, 101–200, 201–500, and 500+ partners. For all other points, the methods of the additional survey were the same as those of the original. The investigation period was five days, from May 17 to 21, 2019 (approximately one year after the original survey). The age composition of the subsequent survey was nearly the same as that of the original (S4 Fig). The additional survey also favored the power-law distribution (see S5 and S7 Figs). The estimates of the power-law exponent tended to be smaller if the raw data was used (see S6 Fig). When the subjects that selected the largest category (500+) were regarded as outliers, the estimated values were close to those of the original study (see S8 Fig). In conclusion, our results did not differ depending on the answer form.

In this study, we focused on the general setting of heterosexual networks. Although this survey is the first study on heterosexual contacts in Japan, there have been many surveys on homosexual contacts in Japan, especially in men who have sex with men (MSM), a topic that has been extensively studied in many cities and countries [22,29]. Data on homosexual contacts are important for predicting the spread of HIV. On the other hand, various STIs, such as syphilis, gonorrhea, hepatitis and HTLV-1, inevitably spread through heterosexual contact. One of the aims of this research was to provide accessible data on the heterosexual network to STI researchers. For example, the current data will be applicable to mathematical models of STIs considering complex networks of heterosexual contacts [30,31]. The value of the basic reproduction number (*R*_0_) depends heavily on the distribution of the number of sexual contacts. Thus, understanding the distribution at the macro level is significant for constructing a mathematical model for STIs.

## Conclusion

We found that the heterosexual network in Japan has a scale-free property. Although some details of the entire network are still unknown, the distribution of the number of sexual contacts provides useful information for developing an effective strategy to reduce the transmission of STIs. Web surveys are useful for the collection of sensitive, private data, such as the number of sexual contacts. Further investigations via Web surveys are needed to understand the universal structure of sexual networks.

## Supporting information

S1 Data The dataset of the 2018 survey on the japanese sexual network. (XLSX)

S2 Data The data set of the 2019 survey on the japanese sexual network. (XLSX)

S1 Fig Age composition. (**A**) and (**B**) show the marital status of survey participants and (**C**) and (**D**) show the number of cumulative sexual partners. (**E**) and (**F**) show the number of sexual partners in the previous three months, and (**A**, **C**, **E**) and (**B**, **D**, **F**) represent males and females, respectively.
(PDF)

S2 Fig Analysis results excluding an outlier. We regarded the woman who reported that she had 999 lifetime partners as outlier. (**A**) shows the cumulative distributions of the number of sexual partners. The red and green curves represent the maximum likelihood fitting of the power-law distribution and the negative binomial distribution, respectively. (**B**) and (**C**) show the model selection using the AIC and BIC to compare the fitting of the power-law distribution (red dots) and the shifted negative binomial distribution (green dots) for the number of sexual partners. (**D**) shows the estimated values of the power-law exponents as a function of *k*_min_. The error bars represent the 95% confidence intervals valuated by the nonparametric (percentile) bootstrap method.
(PDF)

S3 Fig Analysis results excluding an outlier. We regarded the woman who reported that she had 999 lifetime partners in the previous three months as an outlier. (**A**) shows the cumulative distributions of the number of sexual partners. The red and green curves represent the maximum likelihood fitting of the power-law distribution and the negative binomial distribution, respectively. (**B**) and (**C**) show the model selection using the AIC and BIC to compare the fitting of the power-law distribution (red dots) and the shifted negative binomial distribution (green dots) for the number of sexual partners. (**D**) shows the estimated values of the power-law exponents as a function of *k*_min_. The error bars represent the 95% confidence intervals valuated by the nonparametric (percentile) bootstrap method.
(PDF)

S4 Fig Age composition of the respondents of the subsequent web survey. To study the effect of rounding, we conducted a Web survey in which the subjects did not enter the number of sexual partners directly but selected values from the following categories: 1, 2, 3, 4, 5–10, 11–20, 21–50, 51–100, 101–200, 201–500, and 500+ partners. For all other points, the methods of the additional survey were the same as those of the original. The investigation period was five days, from May 17 to 21, 2019 (approximately one year after the original survey). (**A**) and (**B**) show the marital status of the survey participants. (**C**) and (**D**) show the number of cumulative sexual partners. (**E**) and (**F**) show the number of sexual partners in the previous three months, (**A**, **C**, **E**) and (**B**, **D**, **F**) represent males and females, respectively.
(PDF)

S5 Fig Model selection for the subsequent web survey. We use the AIC and BIC to compare the fitting of the power-law distribution (red dots) and the shifted negative binomial distribution (green dots) for the number of sexual partners. The AIC and BIC of the fitting for the number of sexual partners for *k*_min_ = 1,2,3,4,5, and 11; we did not calculate the AIC and BIC for the numbers 6 to 10 because they were in the same category as 5. The results in S5 Fig are essentially unchanged from those in Fig 2, except for *k*_min_ = 11, where the AIC and BIC in S5 Fig (**A–D**) are relatively larger than those in Fig 2**A**–2**D**. The subsequent survey supports the result that the heterosexual network in Japan has a power-law property.
(PDF)

S6 Fig The power-law exponents as a function of *k*_min_ for the subsequent web survey. (**A**) and (**B**) show the cumulative (total) sexual partners for males and females, respectively. (**C**) and (**D**) represent males and females, respectively, in the previous three months. The error bars represent the 95% confidence intervals valuated by the nonparametric (percentile) bootstrap method. The estimated values of the power-law exponent are shown in the bottom list. Although the estimated values of α were slightly less than those in the original survey, tendency for sex and duration was similar.
(PDF)

S7 Fig Model selection for the subsequent web survey excluding outliers. We regarded subjects who reported more than 501 sexual partners as outliers. These plots were obtained in the same way as those in S5 Fig The results in S7 Fig are essentially the same as those in S5 Fig
(PDF)

S8 Fig The power-law exponents as a function of *k*_min_ for the subsequent web survey excluding outliers. We regarded subjects who reported more than 501 sexual partners as outliers. These results were obtained in the same way as those in S6 Fig The estimated values of the power-law exponents are similar to those obtained from the original data (see Table 1).
(PDF)
