# Supplementary material for: Is the network of heterosexual contact in Japan scale free?
Source: PLoS One. 2019 Aug 27;14(8):e0221520. doi: 10.1371/journal.pone.0221520 (PMC6711537; doi:10.1371/journal.pone.0221520)
Supplement: S1 Fig — (A) and (B) show the marital status of survey participants and (C) and (D) show the number of cumulative sexual partners. (E) and (F) show the number of sexual partners in the previous three months, and (A, C, E) and (B, D, F) represent males and females, respectively. (PDF) [file pone.0221520.s003.pdf]

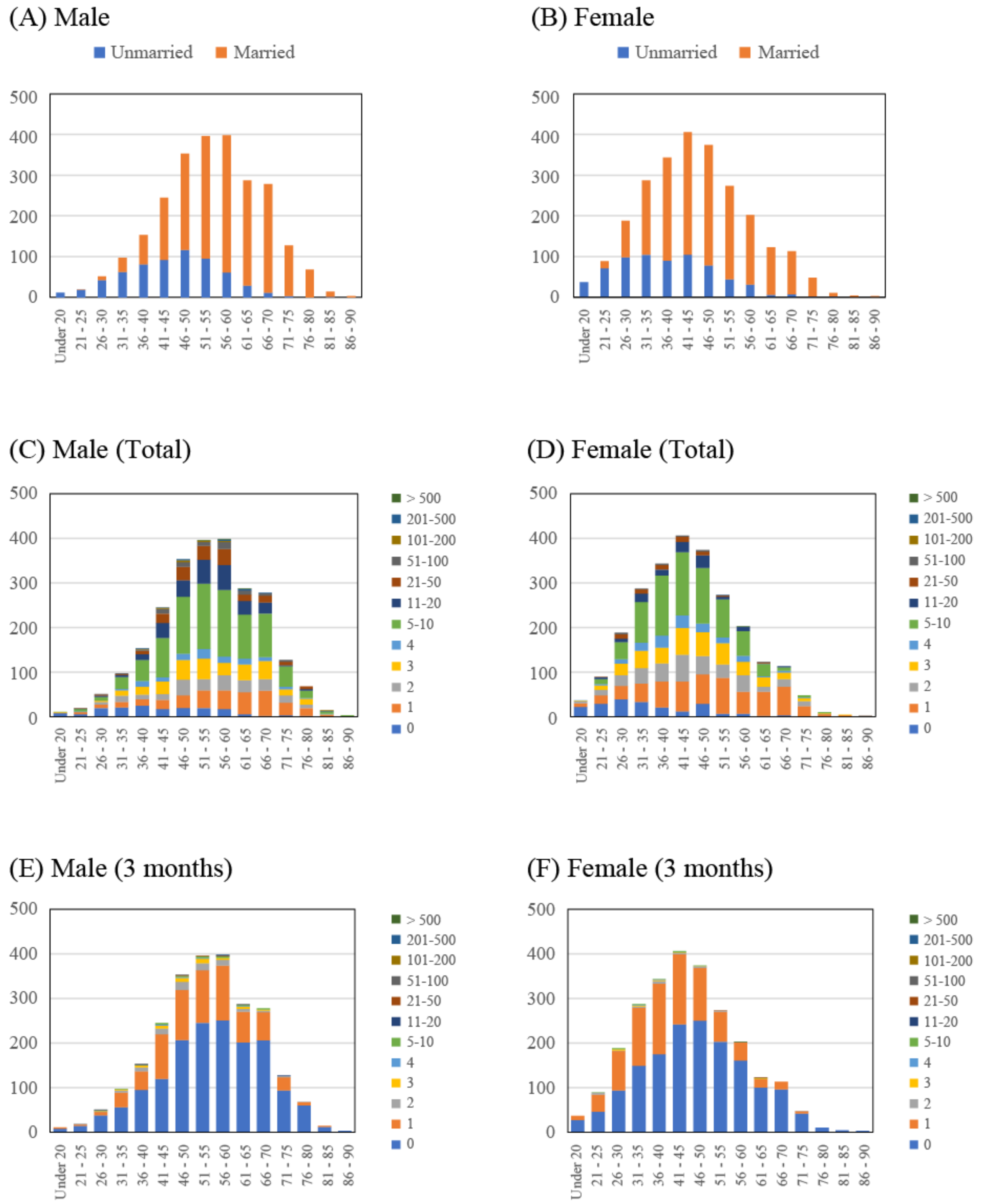

S1 Fig. Age Composition. (**A**) and (**B**) show the marital status of survey participants and (**C**) and (**D**) show the number of cumulative sexual partners. (**E**) and (**F**) show the number of sexual partners in the previous three months, and (**A**, **C**, **E**) and (**B**, **D**, **F**) represent males and females, respectively.
