# Supplementary material for: Is the network of heterosexual contact in Japan scale free?
Source: PLoS One. 2019 Aug 27;14(8):e0221520. doi: 10.1371/journal.pone.0221520 (PMC6711537; doi:10.1371/journal.pone.0221520)
Supplement: S2 Fig — We regarded the woman who reported that she had 999 lifetime partners as outlier. (A) shows the cumulative distributions of the number of sexual partners. The red and green curves represent the maximum likelihood fitting of the power-law distribution and the negative binomial distribution, respectively. (B) and (C) show the model selection using the AIC and BIC to compare the fitting of the power-law distribution (red dots) and the shifted negative binomial distribution (green dots) for the number of sexual partners. (D) shows the estimated values of the power-law exponents as a function of kmin. The error bars represent the 95% confidence intervals valuated by the nonparametric (percentile) bootstrap method. (PDF) [file pone.0221520.s004.pdf]

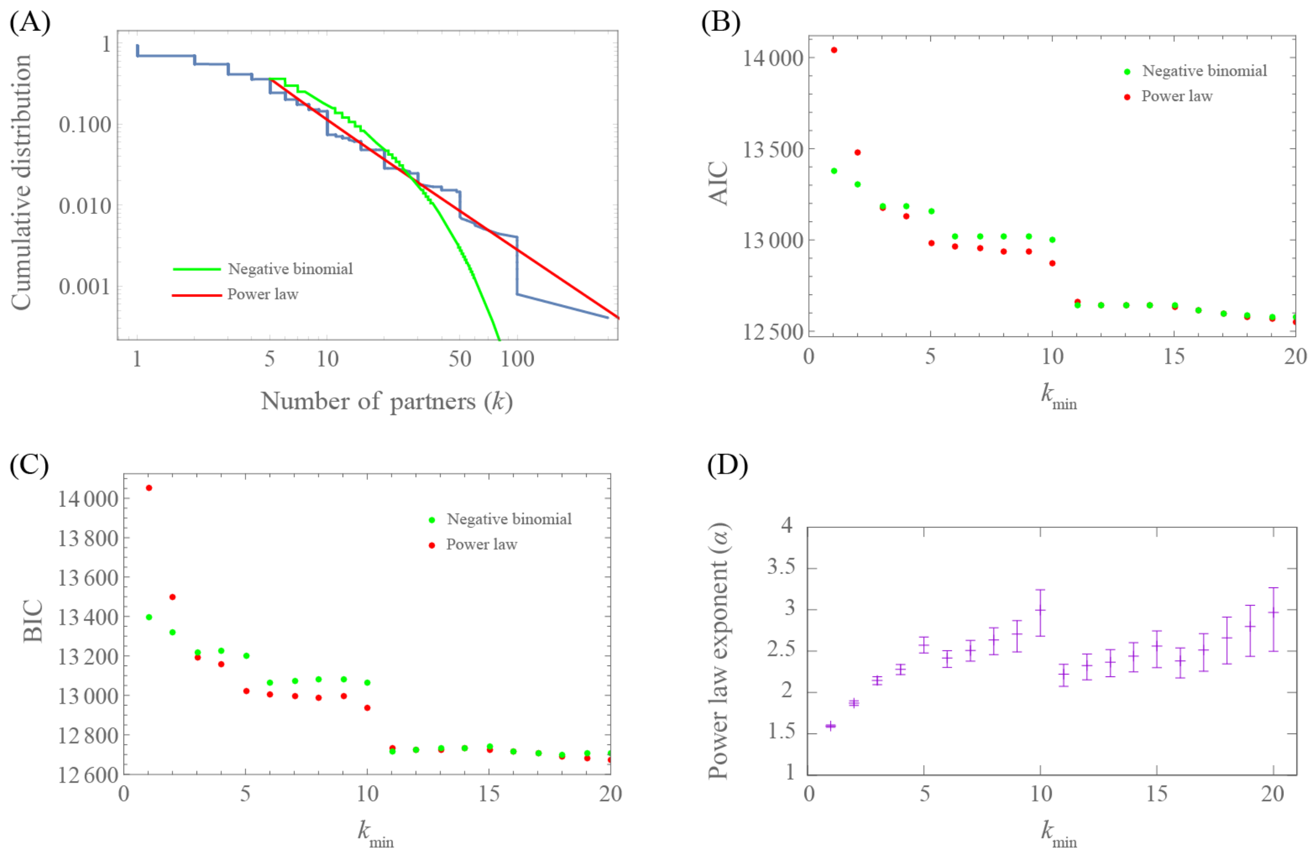

$k_{\min}$	α [(1)]	geo	tpl	α [(2)]
5	2.55	232** (p=0.000)	−0.44* (p=0.35)	2.58 (CI: 2.48-2.67)

S2 Fig. Analysis Results Excluding an Outlier. We regarded the woman who reported that she had 999 lifetime partners as outlier. (**A**) shows the cumulative distributions of the number of sexual partners. The red and green curves represent the maximum likelihood fitting of the power-law distribution and the negative binomial distribution, respectively. (**B**) and (**C**) show the model selection using the AIC and BIC to compare the fitting of the power-law distribution (red dots) and the shifted negative binomial distribution (green dots) for the number of sexual partners. (**D**) shows the estimated values of the power-law exponents as a function of $k_{\min}$. The error bars represent the 95% confidence intervals valuated by the nonparametric (percentile) bootstrap method.
