# Supplementary material for: Is the network of heterosexual contact in Japan scale free?
Source: PLoS One. 2019 Aug 27;14(8):e0221520. doi: 10.1371/journal.pone.0221520 (PMC6711537; doi:10.1371/journal.pone.0221520)
Supplement: S4 Fig — To study the effect of rounding, we conducted a Web survey in which the subjects did not enter the number of sexual partners directly but selected values from the following categories: 1, 2, 3, 4, 5–10, 11–20, 21–50, 51–100, 101–200, 201–500, and 500+ partners. For all other points, the methods of the additional survey were the same as those of the original. The investigation period was five days, from May 17 to 21, 2019 (approximately one year after the original survey). (A) and (B) show the marital status of the survey participants. (C) and (D) show the number of cumulative sexual partners. (E) and (F) show the number of sexual partners in the previous three months, (A, C, E) and (B, D, F) represent males and females, respectively. (PDF) [file pone.0221520.s006.pdf]

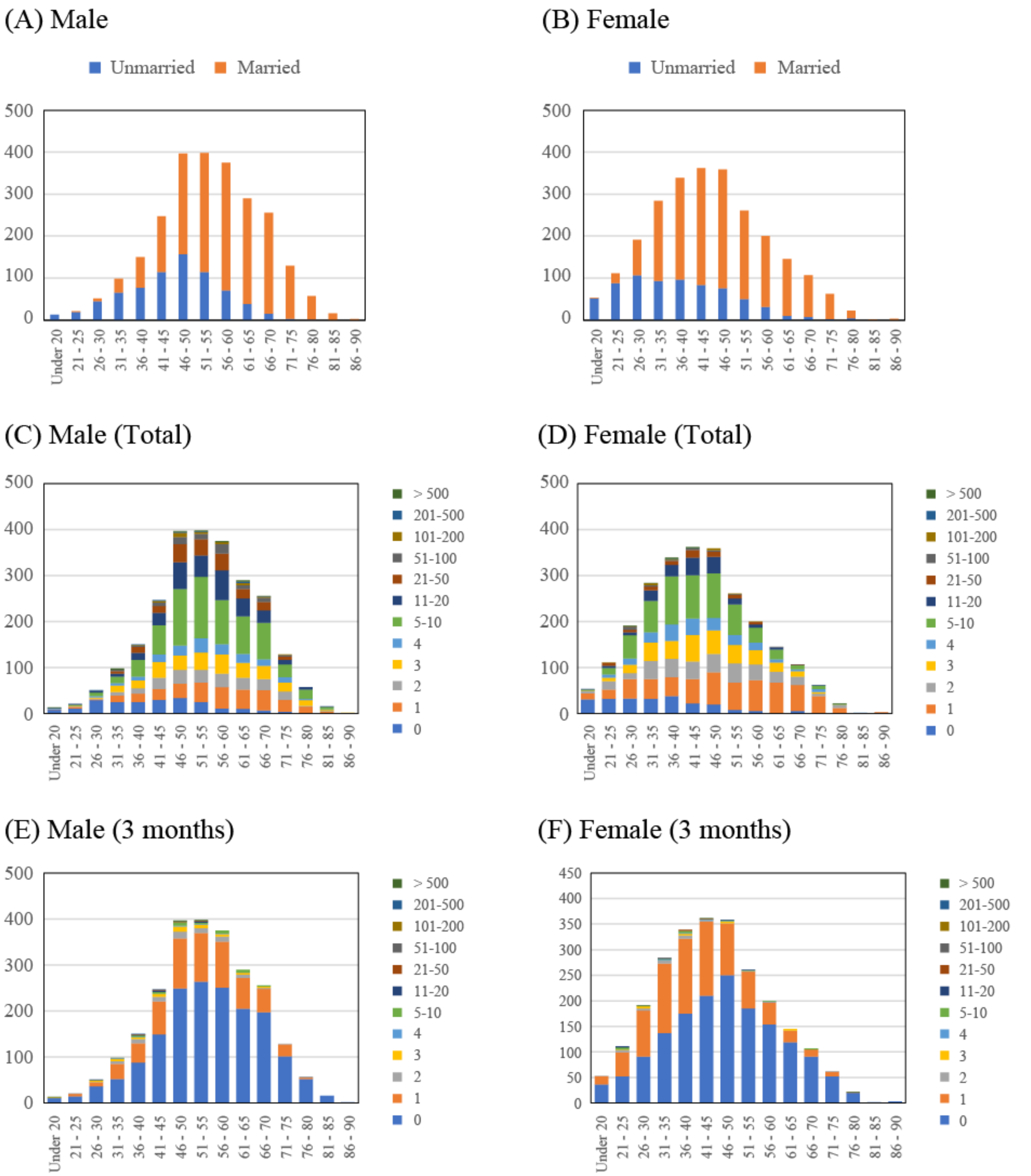

S4 Fig. Age Composition of the Respondents of the Subsequent Web Survey. To study the effect of rounding, we conducted a Web survey in which the subjects did not enter the number of sexual partners directly but selected values from the following categories: 1, 2, 3, 4, 5-10, 11-20, 21-50, 51-100, 101-200, 201-500, and 500+ partners. For all other points, the methods of the additional survey

were the same as those of the original. The investigation period was five days, from May 17 to 21, 2019 (approximately one year after the original survey). (**A**) and (**B**) show the marital status of the survey participants. (**C**) and (**D**) show the number of cumulative sexual partners. (**E**) and (**F**) show the number of sexual partners in the previous three months, (**A**, **C**, **E**) and (**B**, **D**, **F**) represent males and females, respectively.
