# Supplementary material for: Is the network of heterosexual contact in Japan scale free?
Source: PLoS One. 2019 Aug 27;14(8):e0221520. doi: 10.1371/journal.pone.0221520 (PMC6711537; doi:10.1371/journal.pone.0221520)
Supplement: S5 Fig — We use the AIC and BIC to compare the fitting of the power-law distribution (red dots) and the shifted negative binomial distribution (green dots) for the number of sexual partners. The AIC and BIC of the fitting for the number of sexual partners for kmin = 1,2,3,4,5, and 11; we did not calculate the AIC and BIC for the numbers 6 to 10 because they were in the same category as 5. The results in S5 Fig are essentially unchanged from those in Fig 2, except for kmin = 11, where the AIC and BIC in S5 Fig (A–D) are relatively larger than those in Fig 2A–2D. The subsequent survey supports the result that the heterosexual network in Japan has a power-law property. (PDF) [file pone.0221520.s007.pdf]

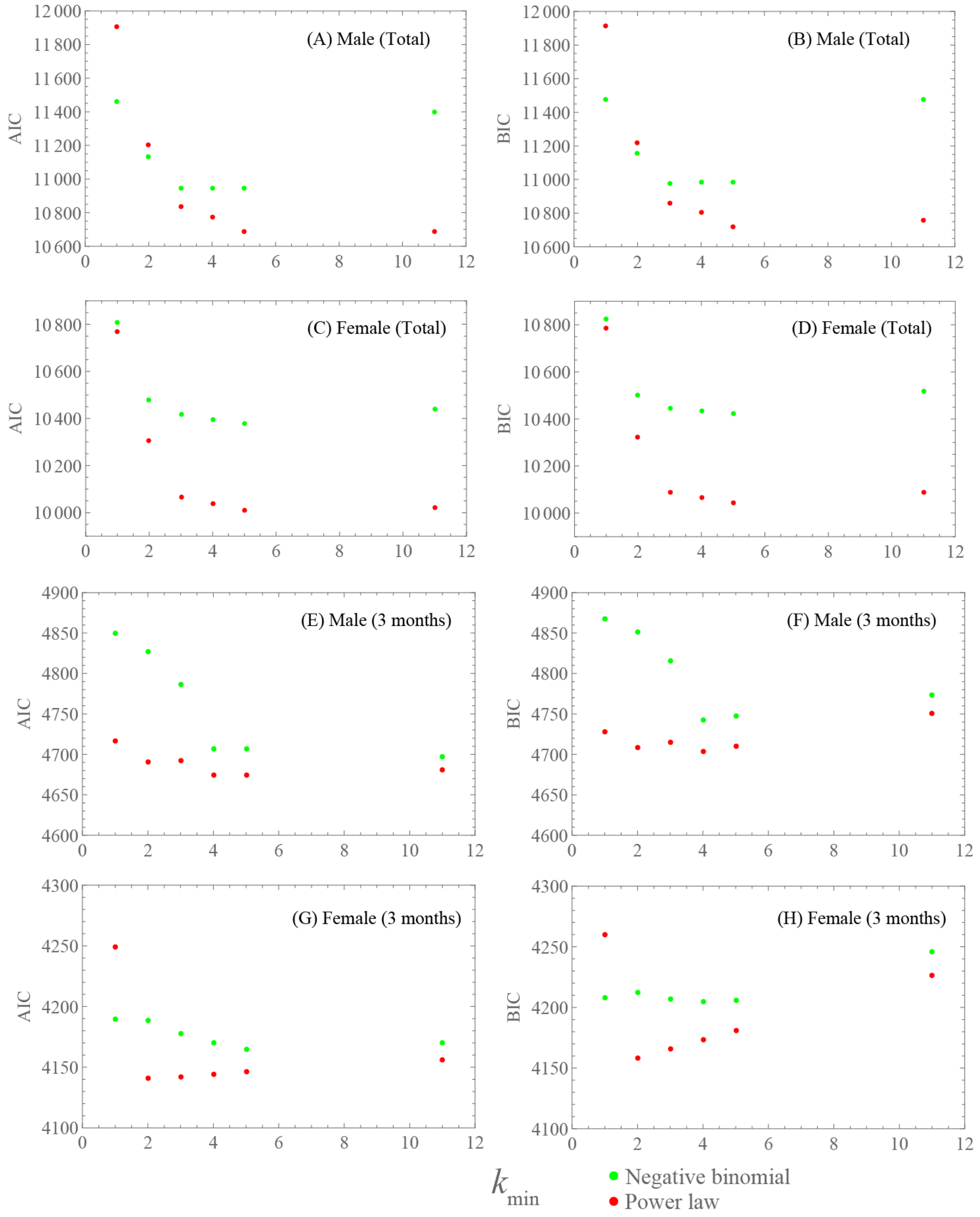

S5 Fig. Model Selection for the Subsequent Web Survey. We use the AIC and BIC to compare the fitting of the power-law distribution (red dots) and the shifted negative binomial distribution (green

dots) for the number of sexual partners. The AIC and BIC of the fitting for the number of sexual partners for $k_{\min} = 1, 2, 3, 4, 5, \text{and } 11$; we did not calculate the AIC and BIC for the numbers 6 to 10 because they were in the same category as 5. The results in S5 Fig are essentially unchanged from those in Fig 2, except for $k_{\min} = 11$, where the AIC and BIC in S5 Fig. (**A-D**) are relatively larger than those in Fig 2 (**A-D**). The subsequent survey supports the result that the heterosexual network in Japan has a power-law property.
