# Supplementary material for: Is the network of heterosexual contact in Japan scale free?
Source: PLoS One. 2019 Aug 27;14(8):e0221520. doi: 10.1371/journal.pone.0221520 (PMC6711537; doi:10.1371/journal.pone.0221520)
Supplement: S6 Fig — (A) and (B) show the cumulative (total) sexual partners for males and females, respectively. (C) and (D) represent males and females, respectively, in the previous three months. The error bars represent the 95% confidence intervals valuated by the nonparametric (percentile) bootstrap method. The estimated values of the power-law exponent are shown in the bottom list. Although the estimated values of α were slightly less than those in the original survey, tendency for sex and duration was similar. (PDF) [file pone.0221520.s008.pdf]

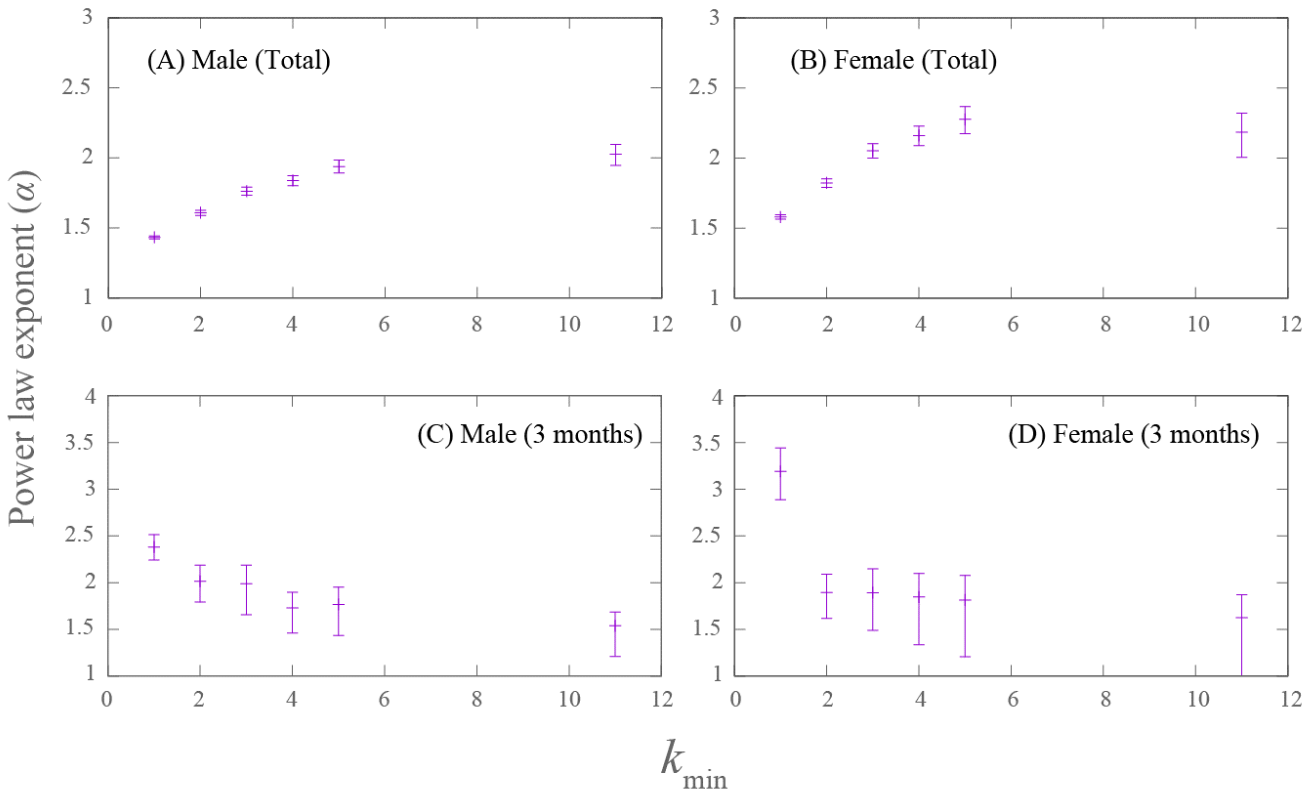

	$k_{\min}$	α
Male (lifetime)	11	2.02 (CI: 1.94-2.10)
Female (lifetime)	5	2.28 (CI: 2.17-2.37)
Male (3 months)	2	2.02 (CI: 1.79-2.19)
Female (3 months)	2	1.90 (CI: 1.66-2.09)

S6 Fig. The Power-law Exponents as a Function of $k_{\min}$ for the Subsequent Web Survey. (**A**) and (**B**) show the cumulative (total) sexual partners for males and females, respectively. (**C**) and (**D**) represent males and females, respectively, in the previous three months. The error bars represent the 95% confidence intervals valuated by the nonparametric (percentile) bootstrap method. The estimated values of the power-law exponent are shown in the bottom list. Although the estimated values of α were slightly less than those in the original survey, tendency for sex and duration was similar.
