# Supplementary material for: Is the network of heterosexual contact in Japan scale free?
Source: PLoS One. 2019 Aug 27;14(8):e0221520. doi: 10.1371/journal.pone.0221520 (PMC6711537; doi:10.1371/journal.pone.0221520)
Supplement: S7 Fig — We regarded subjects who reported more than 501 sexual partners as outliers. These plots were obtained in the same way as those in S5 Fig The results in S7 Fig are essentially the same as those in S5 Fig (PDF) [file pone.0221520.s009.pdf]

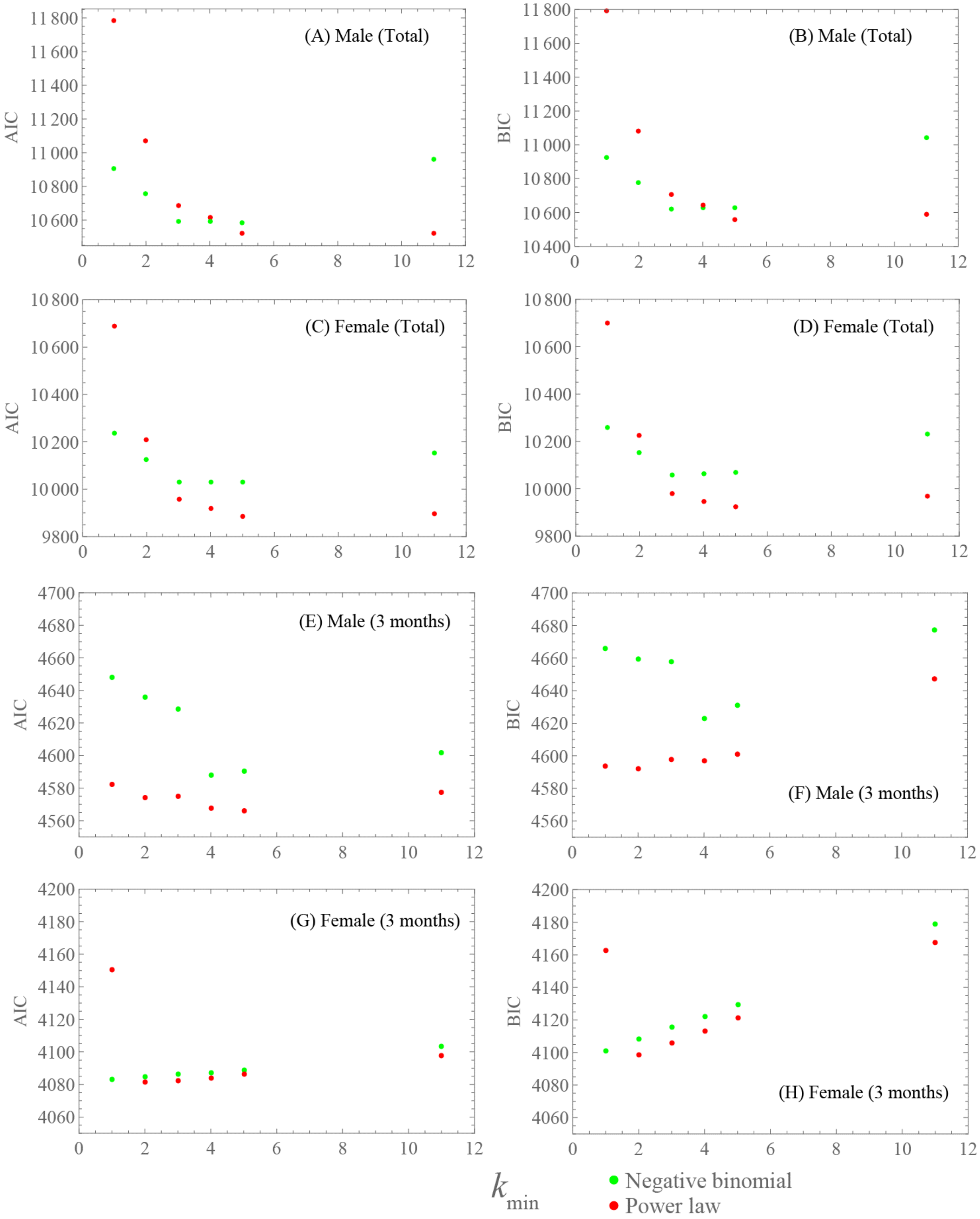

(A) Male (Total)
(B) Male (Total)
(C) Female (Total)
(D) Female (Total)
(E) Male (3 months)
(F) Male (3 months)
(G) Female (3 months)
(H) Female (3 months)
AIC
BIC
k_{min}
Negative binomial
Power law

S7 Fig. Model Selection for the Subsequent Web Survey Excluding Outliers. We regarded subjects who reported more than 501 sexual partners as outliers. These plots were obtained in the same way as those in S5 Fig. The results in S7 Fig are essentially the same as those in S5 Fig.
