# Supplementary material for: Is the network of heterosexual contact in Japan scale free?
Source: PLoS One. 2019 Aug 27;14(8):e0221520. doi: 10.1371/journal.pone.0221520 (PMC6711537; doi:10.1371/journal.pone.0221520)
Supplement: S8 Fig — We regarded subjects who reported more than 501 sexual partners as outliers. These results were obtained in the same way as those in S6 Fig The estimated values of the power-law exponents are similar to those obtained from the original data (see Table 1). (PDF) [file pone.0221520.s010.pdf]

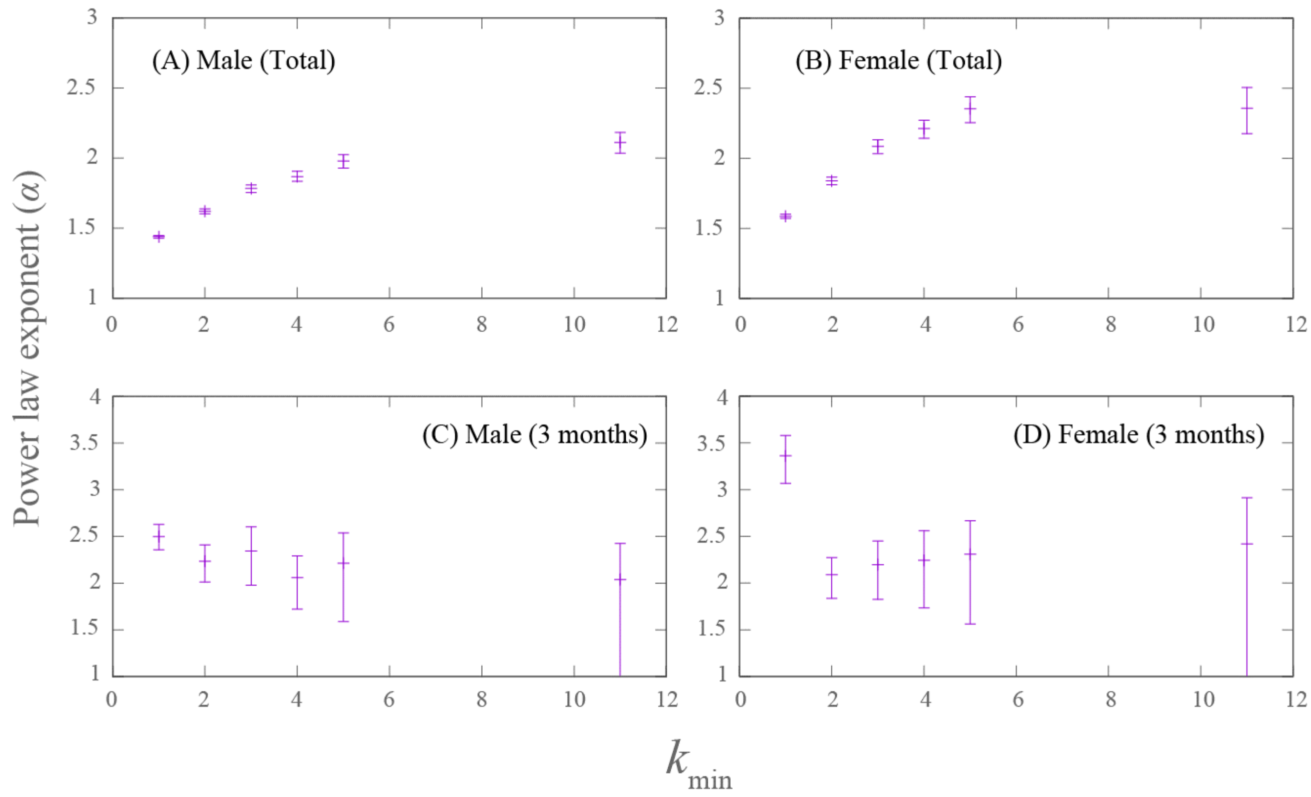

	k_{min}	α
Male (lifetime)	11	2.11 (CI: 2.03-2.18)
Female (lifetime)	5	2.35 (CI: 2.25-2.44)
Male (3 months)	2	2.23 (CI: 2.01-2.40)
Female (3 months)	2	2.09 (CI: 1.84-2.27)

S8 Fig. The Power-law Exponents as a Function of k_{min} for the Subsequent Web Survey Excluding Outliers. We regarded subjects who reported more than 501 sexual partners as outliers. These results were obtained in the same way as those in S6 Fig. The estimated values of the power-law exponents are similar to those obtained from the original data (see Table 1).
